# CD38 Deficiency Downregulates the Onset and Pathogenesis of Collagen-Induced Arthritis through the NF-*κ*B Pathway

**DOI:** 10.1155/2019/7026067

**Published:** 2019-03-05

**Authors:** Yuna Du, Qianqian Dai, Huiqing Zhang, Qi Li, Kuangyu Song, Yingyuan Fu, Weiping Min, Zhenlong Liu, Rong Li

**Affiliations:** ^1^Department of Medical Microbiology & Immunology and Laboratory of Infection & Immunity, School of Basic Medical Sciences, Nanchang University, Nanchang, China; ^2^Jiangxi Provincial Key Laboratory of Immunotherapy, Nanchang, China; ^3^University of Western Ontario, Departments of Surgery, Pathology, and Oncology, London, Canada; ^4^McGill University, Division of Experimental Medicine, Department of Medicine, Montreal, Quebec, Canada

## Abstract

**Aim:**

The RelB gene plays an important role in guiding the progression of arthritis. We have previously demonstrated that the expression of the RelB gene is decreased significantly in bone marrow DCs of CD38^−/−^ mice. In this study, we demonstrate that the cluster of the differentiation (CD38) gene could be a potentially therapeutic target for autoimmune arthritis.

**Method:**

Collagen-induced arthritis (CIA) models were generated with both the wild-type (WT) C57BL/6 and CD38^−/−^ mice. The expression of the RelB gene and maturation of bone marrow-derived dendritic cells (DCs) from the WT and CD38^−/−^ mice were detected. Antigen-specific T cell responses, joint damage, and expression of proinflammatory cytokines were assessed. The effects of the Nuclear Factor Kappa B (NF-*κ*B) transcription factor and its mechanisms were characterized.

**Results:**

We demonstrated that in CD38^−/−^ mice, the expression of the RelB gene and major histocompatibility complex II (MHC II) was decreased, accompanied with the inhibited T cell reaction in a mixed lymphocyte reaction (MLR) in bone marrow-derived DCs. Compared to the serious degeneration of the cartilage and the enlarged gap of the cavum articular in WT CIA mice, joint pathological changes of the CD38^−/−^ CIA mice revealed marked attenuation, while the joint structures were well preserved. The preserved effects were observed by the inhibition of proinflammatory cytokines and promotion of anti-inflammatory cytokines. Furthermore, decreased phosphorylation of NF-*κ*B was also observed in CD38^−/−^ CIA mice.

**Conclusion:**

We demonstrate that CD38 could regulate CIA through NF-*κ*B and this regulatory molecule could be a novel target for the treatment of autoimmune inflammatory joint disease.

## 1. Introduction

Rheumatoid arthritis (RA) is a chronic inflammatory disease and afflicts 1% of the world's population [1]. The characteristic of RA is nonspecific, symmetric inflammation of the joint accompanied with destruction of the cartilage and bone [2]. RA synovial fibroblasts (RASFs) are a specialized cell type which is located in synovial joints [1, 3]. When activated, RASFs can produce proinflammatory cytokines, such as TNF-*α* and IL-1*β* [4, 5] and play an important role in both initiation and development of RA [6]. Disease-Modifying Antirheumatic Drug (DMARD) therapy has a greater beneficial impact on the RA outcome [7, 8], but none of the currently available treatments provide a drug-free and long-lasting remission of RA [9]. Moreover, serious side effects such as infections were shown in some patients [10].

Autoimmunity can be prevented by active silencing of autoreactive T cells and inhibiting the central role of DCs. Because of the important role of DCs in adjusting adaptive immune responses, current immunotherapeutic approaches aim at achieving restoration of immune tolerance by treatment with tolerogenic DCs (Tol-DCs) [11]. Generally, immature DCs act primarily as tolerogenic cells: they can promote the generation of T regulatory cells and cause deviation of cytokines from Th1 to Th2, whereas mature DCs act as immune stimulators [12]. RelB, a member of the NF-*κ*B family, plays an important role in the maturation and functions of dendritic cells [4, 13, 14]. And the antigen-specific T cell responses were blunted in both RelB-silenced DCs [13] and splenic DCs of RelB mutant mice [14]. We have previously demonstrated in the animal model of collagen-induced arthritis (CIA) that treatment with a synthetic RelB inhibitor or RelB gene-silenced Tol-DCs can prevent disease progression. The decreased expression of maturation markers, i.e., CD40, CD80, and CD86 in those Tol-DCs, was confirmed [4, 15].

On the other hand, CD38 is a cyclic ADP ribose hydrolase glycoprotein with molecular weight of 42 kD, and CD38 is located on the cell surface with a variety of functions: CD38 can transduce the intracellular proliferative signals and ectoenzymatic activity related to the catabolism of extracellular nucleotides [16]. CD38 plays a variety of regulatory effects on cardiovascular diseases, metabolic diseases, and tumors [17]. But, the role of CD38 in autoimmune diseases remain largely unknown.

Our previous data showed that the expression of the RelB gene significantly decreased in bone marrow DCs (BMDCs) of CD38^−/−^ mice. Thus, this study aims at investigating the effects of the CD38 gene on the development of RA and at exploring the underlying molecular mechanism.

## 2. Materials and Methods

### 2.1. Animals

The male C57BL/6 (WT), BALB/c, and CD38^−/−^ mice were obtained from The Jackson Laboratory (Bar Harbor, ME) and maintained in sterile filter-top cages in SPF Animal Facility at Laboratory Animal Center of Nanchang University. The experimental procedures on use and care of animals had been approved by the Ethics Committee of Nanchang University, and the ethical approval number is SYXK 2015-0001. All animals were used at the age of 8 weeks.

### 2.2. PCR

Tails (1 cm) were taken from the WT and CD38^−/−^ mice, respectively, and treated with a 400 *μ*l lysis buffer and 10 *μ*l proteinase K (20 mg/ml). After being incubated overnight at 56°C, DNA was extracted with a quick genotyping assay kit for the mouse tail (Beyotime). Polymerase chain reaction (PCR) was performed in a 13 *μ*l of reaction volume containing 0.2 *μ*M primers and 1 U Taq DNA polymerase under the following conditions: 94°C for 30 sec, 60°C for 30 sec, and then 72°C for 30 sec (30 cycles). PCR products were visualized with Safe Green on 1.5% agarose gels. Primers used for the amplification of murine CD38, Neomycin- (Neo-) resistant gene, and GAPDH were as follows: CD38, 5′-CAATGTCCCAATCTGCCAAG-3′ (forward); Neo, 5′-GCTGCGATTCGGGAGGGATAC-3′ (forward); both CD38 and Neo, 5′-AAAGGGGAGAACAGGAAGGA (reverse); and GAPDH, 5′-GAAGGTGGTGAAGCAGGCATC-3′ (forward) and 5′-GTGGGAGTTGCTGTTGAAGTCG-3′ (reverse).

### 2.3. CIA Model

The male WT and CD38^−/−^ mice, 8 weeks of age, were immunized (day 0) intradermally at the base of the tail with 100 *μ*g/100 *μ*l chicken type II collagen (CII) (Sigma-Aldrich, St. Louis, MO) and mixed with 500 *μ*g/100 *μ*l of complete Freund's adjuvant (CFA) (Sigma-Aldrich). On day 21, after priming, the mice received an intradermal booster injection with the same mixture of CII with CFA. Mice were examined visually three times per week by the double-blind method, and the different appearance of peripheral joints were observed.

### 2.4. DC Cultures

Bone marrow cells were flushed from the tibias and femurs of the WT and CD38^−/−^ mice, respectively, on day 0, and 4 × 10^6^ cells/well were cultured in 6-well plates (Corning, NY) in 4 ml of a complete medium (RPMI 1640 supplemented with 2 mM L-glutamine, 100 U/ml penicillin, 100 *μ*g of streptomycin, and 10% FCS (Invitrogen, USA) supplemented with recombinant mouse IL-4 (10 ng/ml; PeproTech) and recombinant GM-CSF (10 ng/ml; PeproTech)). All cultures were incubated at 37°C in 5% humidified CO_2_. 48 hrs later, nonadherent cells were removed and a fresh medium was added every 48 hrs [7].

### 2.5. RT-qPCR

Total RNA was extracted from cells using Trizol (Invitrogen). cDNA was synthesized with 1 *μ*g total RNA, oligdT, and reverse transcriptase (Invitrogen) in 20 *μ*l reaction volume. Primers used for the amplification of murine RelB, IL-1*β*, TNF-*α*, IL-4, IL-10, and GAPDH were as follows: RelB, 5′-AATGCTGGCTCCCTGAAGAACC-3′ (forward) and 5′-ATGTCCCTGCTGGTCCCGATAG-3′ (reverse); IL-1*β*, 5′-TTTTCCTCCTTGCCTCTGAT-3′ (forward) and 5′-GAGTGCTGCCTAATGTCCCC-3′ (reverse); TNF-*α*, 5′-AGCCGATGGGTTGTACCTTG-3′ (forward) and 5′-GTGGGTGAGGAGCACGTAGTC-3′ (reverse); IL-4, 5′-AGCTAGTTGTCATCCTGCTCTTCT-3′ (forward) and 5′-CGAGTAATCCATTTGCATGATGCT-3′ (reverse); IL-10, 5′-GCTCTTACTGACTGGCATGAG-3′ (forward) and 5′-CGCAGCTCTAGGAGCATGTG-3′ (reverse); and GAPDH, 5′-GAAGGTGGTGAAGCAGGCATC-3′ (forward) and 5′-GTGGGAGTTGCTGTTGAAGTCG-3′ (reverse). Quantitative real-time PCR was performed in 20 *μ*l of reaction volume containing 1 U Taq DNA polymerase and 0.2 *μ*mol/l primers under PCR conditions consisted of 30 sec at 95°C followed by 40 cycles of 5 sec 95°C and 30 sec at 60°C.

### 2.6. FACS Staining and Analysis of BMDCs

DCs were generated from bone marrow progenitor cells of the WT and CD38^−/−^ mice as previously described [7]. Seven days after culture, DCs were harvested and stained with anti-CD11C-Per CP (0.5 *μ*g/test), anti-MHC II FITC (0.5 *μ*g/test), anti-CD40-PE (0.125 *μ*g/test), and anti-CD80 APC (0.06 *μ*g/test) antibodies (Bioscience, USA). Flow cytometry analysis was performed with FlowJo software in a FACSCanto II (BD Biosciences) system.

### 2.7. Mixed Leukocyte Reaction (MLR)

10 days after CII booster injection, DCs were generated from bone marrow progenitor cells of the WT and CD38^−/−^ mice as described above. Seven days after culture, DCs from 2 groups were seeded in a flat-bottom 96-well plate (Corning) for use in triplicate as stimulator cells. Spleen T cells from BALB/c mice were isolated with Ficoll-Paque (GE Healthcare Bio-sciences AB) by gradient centrifugation and added as responders (5 × 10^5^ cells/well). The mixed lymphocytes were cultured in 200 *μ*l of RPMI 1640 (supplemented with 10% FCS, 100 *μ*g/ml of streptomycin, and 100 U/ml of penicillin) at 37°C for 72 hrs. 72 hrs after incubation, 20 *μ*l CCK-8 was added to each well for 2 hrs. The OD of each well was measured in an ELISA plate reader at a wavelength of 450 nm.

### 2.8. Histology

The paws from the normal WT, WT CIA, and CD38^−/−^ CIA mice were removed, and joint tissues were fixed in 10% (wt/vol) neutral-buffered formalin in 0.15 M PBS (pH 7.4). Joint tissues were decalcified with decalcifier solution for 2 weeks and accompanied with dehydration in a gradient of alcohols subsequently; tissues were processed for paraffin embedding in paraplast by classic procedure. Serial paraffin sections throughout the joint were cut at 7 *μ*m thickness on a microtome, heated at 60°C for 30 min, and deparaffinized. Hydration was done by transferring the sections through the following solutions: triple to xylene for 6 min and then twice to 100% ethanol twice, 95% ethanol, and 70% ethanol for 2 min. Sections were stained with H&E and mounted on glass slides.

### 2.9. Western Blot

DCs were generated from bone marrow progenitor cells of the WT and CD38^−/−^ CIA or healthy mice as described above 5 weeks after CII booster injection. After 7 days of culture, cells were harvested, washed twice with ice-cold PBS, resuspended in the protein lysis buffer with a protease inhibitor, and then kept on ice for 30 min. Lysed cells were centrifuged at 15000 rpm for 20 min at 4°C; the supernatant was collected and preserved at -80°C for future use. Protein concentration was determined by Bio-Rad protein assay, and 30 *μ*g lysate was separated on 12% SDS-PAGE; transferred to a nitrocellulose membrane; blocked with 5% fat-free milk and 3% BSA in TBST, anti-NF-*κ*B p65 Rabbit mAb (1 : 1000), anti-Phospho-NF-*κ*B p65 Rabbit mAb (1 : 500), anti-NF-*κ*B1 p105/p50 Rabbit mAb (1 : 1000), anti-Phospho-NF-*κ*B p105 Rabbit mAb (1 : 500), anti-RelB Rabbit mAb (1 : 1000), and anti-GAPDH mAb (1 : 5000) (CST, USA) according to the manufacturer's instructions; and visualized by an ECL assay (Sage Creation).

### 2.10. Statistical Analysis

Data are expressed as mean ± SEM. Differences between groups of mice were compared using the *t*-test for parametric data. A *P* value less than 0.05 was considered significant.

## 3. Results

### 3.1. Maturation of DCs Inhibited by the Knockout of CD38 *In Vivo*

**Figure 1:**
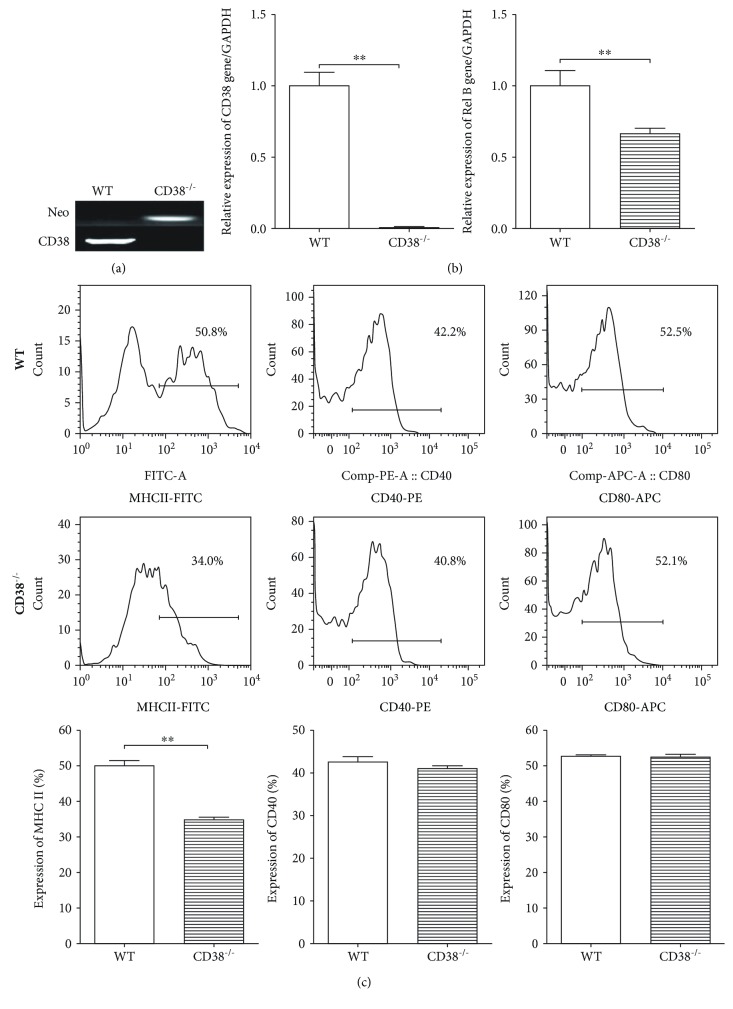
Alteration of the RelB gene expression and phenotype of BMDCs generated over 7 days from CD38^−/−^ mice. (a) Genotyping of CD38 gene deficiency in CD38^−/−^ mice (*n* = 3/group). DNA was extracted from tails of CD38^−/−^ mice as described in Materials and Methods. The CD38 and Neo gene expressions were determined by PCR. (b) Alteration of RelB gene expression in DCs from CD38^−/−^ mice (*n* = 3/group). DCs were cultured from the bone marrow of the WT and CD38^−/−^ mice as described in Materials and Methods. Gene expressions of CD38 and RelB were detected by RT-qPCR. (c) Phenotypes of DCs in CD38^−/−^ mice (*n* = 3/group). DCs were stained with antibodies against MHC II, CD40, and CD 80, respectively; the expression of above molecules was detected by flow cytometry. The data presented one of three independent experiments (^∗∗^*p* < 0.01).

It has been reported that Tol-DCs play an important role in suppressing immune responses [9]. RelB is a main factor controlling the maturation and function of DCs [4]. In this study, we used CD38^−/−^ mice in which genotyping showed the expression of the CD38 gene is deficient while the expression of the Neo gene is positive (Figure 1(a)). The DCs generated from CD38^−/−^ mice also demonstrated that the expression of the CD38 gene is deficient (Figure 1(b)). Interestingly, the expression of the RelB gene in DCs of CD38^−/−^ mice is decreased significantly, when compared to WT mice (*P* < 0.05) (Figure 1(b)).

We previously reported that silencing of the RelB gene in bone marrow-derived DCs can enhance tolerogenic properties [4]. To test if the decreased expression of the RelB gene in CD38^−/−^ DCs is associated with DC maturation, we detected the DC maturation markers MHC II, CD40, and CD80 on DCs of CD38^−/−^ mice. We found that the expression of MHC II is decreased significantly (*P* < 0.01) (Figure 1(c)). Taken together, these data suggest that the maturation of DCs in CD38^−/−^ mice was inhibited accompanied with the repression of RelB.

### 3.2. Immunorepressed Antigen Presentation Ability of BMDCs in CD38^−/−^ Mice

**Figure 2:**
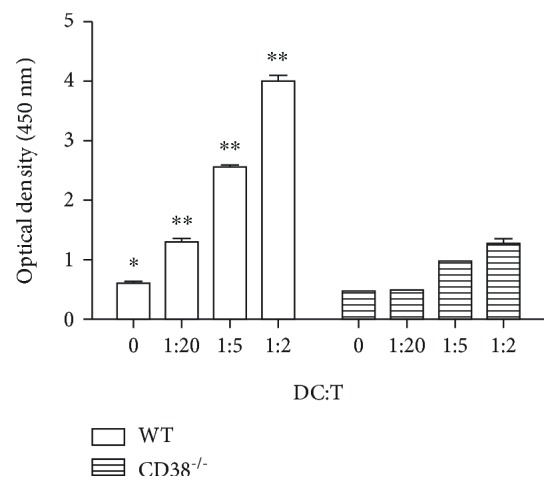
Inhibition of allogeneic stimulatory function of BMDCs generated over 7 days from CD38^−/−^ mice. DCs cultured from the bone marrow of the WT and CD38^−/−^ mice (*n* = 3/group, DCs in each group were combined and distributed into 3 wells independently) were used as stimulator cells and incubated with allogeneic T cells from BALB/c mice for 3 days in an MLR. T cell proliferation was detected by CCK-8 assay. The data presented one of three independent experiments (^∗^*p* < 0.05, ^∗∗^*p* < 0.01).

Our previous data show that silencing IL-12 or the RelB gene in DCs inhibited the antigen presentation ability of DCs. At the meantime, Tol-DCs have low Ag-specific T cell recall responses. To test DC function, we assessed the MLR using BMDCs from the WT or CD38^−/−^ mice with allogeneic T cells from BALB/c mice. MLR in which there was stimulation by CD38^−/−^ BMDCs showed impaired T cell proliferations (Figure 2).

### 3.3. Attenuation of Joint Damage in CD38^−/−^ CIA Mice

**Figure 3:**
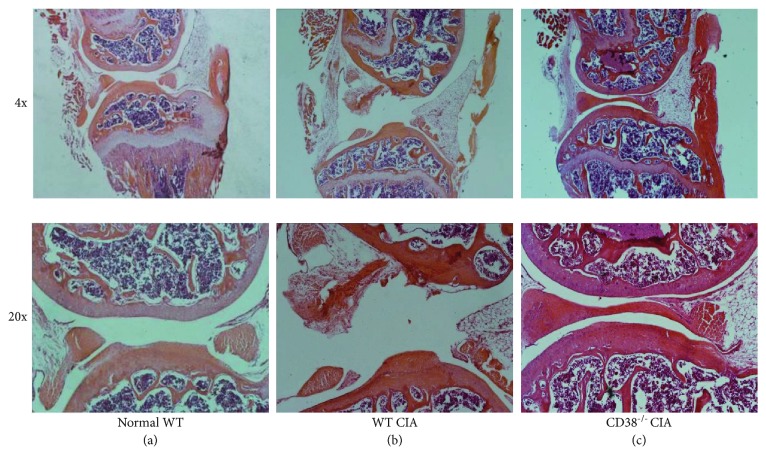
Attenuated joint damage in CD38^−/−^ CIA mice. CIA mice were generated from the WT and CD38^−/−^ mice, as described in Materials and Methods. The knees of mice were collected from the normal WT, WT CIA, and CD38^−/−^ CIA mice at the end point (5 weeks postboosting of the CII antigen). Histological sections of joints were stained with H&E. Pathological changes in the joints of normal WT mice (a), WT CIA mice (b), and CD38^−/−^ CIA mice (c) are displayed (*n* = 3 per group/experiment).

Joint damage in RA results in adverse effects on cartilage degeneration and bone remodeling [1, 18]. To confirm the modulatory effect of CD38 on joint damage in CIA, we further sought to examine microscopic histological differences in CD38^−/−^ CIA mice. CIA mice were sacrificed 5 weeks following the onset of arthritis, and joints were examined by serial sectioning. We observed that, compared with normal mice (Figure 3(a)), CIA mice possessed serious degradation of the cartilage and presented the enlarged cavum articular (Figure 3(b)). In contrast, joint pathological changes of the CD38^−/−^ CIA mice revealed noticeable attenuation although the joint structures were well preserved (Figure 3(c)). These data imply that CD38 deficiency is beneficially associated in the context of the onset and pathogenesis of CIA.

### 3.4. Suppression of Proinflammatory Cytokines in BMDCs Generated over 7 Days from CD38^−/−^ CIA Mice

**Figure 4:**
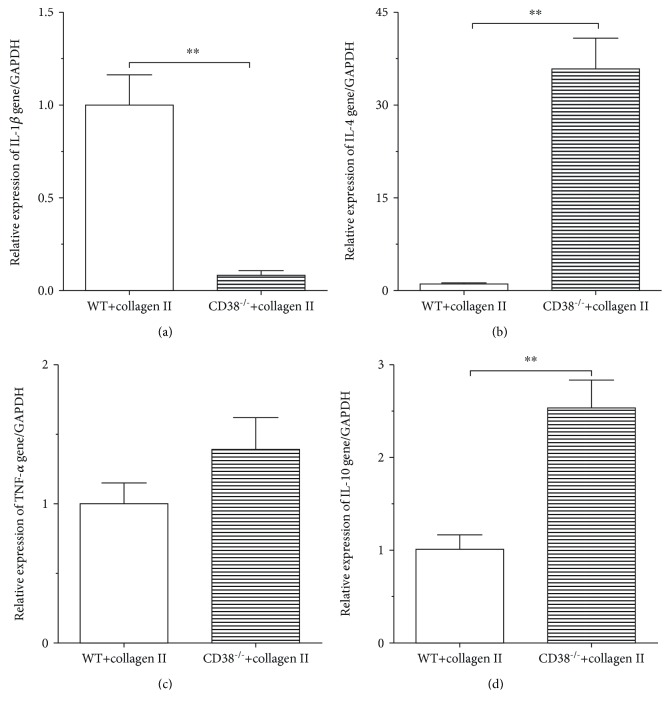
Alteration of proinflammatory cytokines in BMDCs generated over 7 days from CD38^−/−^ CIA mice. DCs were cultured from the bone marrow of the WT and CD38^−/−^ CIA mice, and the mRNA was extracted from the WT and CD38^−/−^ DCs. The expressions of IL-1*β* (a), TNF-*α* (b), IL-4 (c), and IL-10 (d) were detected by RT-qPCR. Results show average levels of those genes expressed as OD (*n* = 3 per group/experiment) (^∗∗^*p* < 0.01).

There are some evidences showing that proinflammatory cytokines play important roles in the onset and progression of autoimmune arthritis [9, 18]. To assess if CD38 can influence the expression of cytokines in CIA mice, we tested cytokine production of BMDCs isolated from the WT and CD38^−/−^ CIA mice. As shown in Figure 3, the mRNA level of IL-1*β* was considerably decreased, although there is no significant change of TNF-*α* transcripts. Compared with BMDCs from WT CIA mice, mRNA levels of IL-4 and IL-10 were significantly increased in CD38^−/−^ CIA mice (Figures 4(a)–4(d)). These data also give us a hint that the inflammatory reaction might be suppressed when CD38 is deficient.

### 3.5. Reduced Phosphorylation of NF-*κ*B in BMDCs from CD38^−/−^ CIA Mice

**Figure 5:**
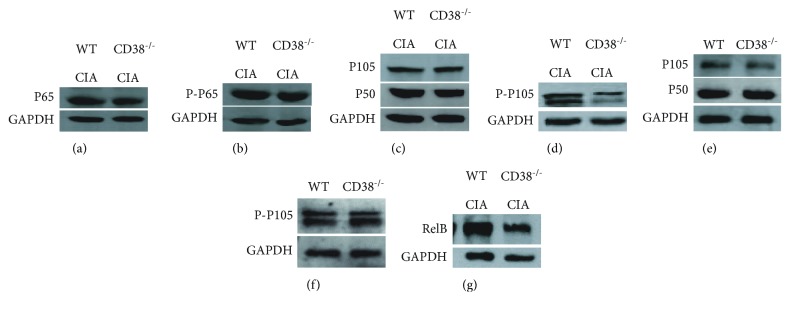
Decreased phosphorylation of NF-*κ*B in BMDCs generated over 7 days from CD38^−/−^ CIA mice. The protein was extracted from DCs in the WT and CD38^−/−^ mice with or without collagen stimuli. Expressions of NF-*κ*B p65 (a), Phospho-NF-*κ*B p65 (b), NF-*κ*B1 p105 (c), Phospho-NF-*κ*B1 p105 (d), and RelB (g) in WT CIA and CD38^−/−^ CIA mice were detected by Western blot (*n* = 3 per group/experiment). Expressions of NF-*κ*B1 p105 (e) and Phospho-NF-*κ*B1 p105 (f) in the WT and CD38^−/−^ healthy mice were also detected by Western blot as normal control (*n* = 3 per group/experiment).

NF-*κ*B is an important regulator of inflammation and immune response [19]. Activation of NF-*κ*B influences the expression of proinflammatory genes, such as growth factors, cytokines, and chemokines [20]. To explore the mechanisms whether CD38 gene deficiency can reduce the expression of proinflammatory genes, we detected the expression of NF-*κ*B and their phosphorylation. Five weeks following the onset of arthritis, BMDCs generated over 7 days were isolated from the WT and CD38^−/−^ mice with or without collagen stimuli, and the expression of NF-*κ*B in BMDCs was detected. Our results show that NF-*κ*B p65 (RelA) (Figure 5(a)), NF-*κ*B P-p65 (Figure 5(b)), and NF-*κ*B1 p105 (Figure 5(c)) were not changed in CD38^−/−^ CIA mice compared to WT CIA mic and that the phosphorylation of NF-*κ*B1 p105 is greatly decreased in CIA mice when the CD38 gene is deficient (Figure 5(d)), whereas, compared to WT mice, the phosphorylation of NF-*κ*B1 p105 in healthy CD38^−/−^ mice was not changed (Figures 5(e) and 5(f)). In addition, RelB (Figure 5(g)) was greatly decreased in BMDCs of CD38^−/−^ CIA mice, which means NF-*κ*B1 p105 phosphorylation and RelB expression were reduced in CD38^−/−^ CIA mice.

## 4. Discussion

Rheumatoid arthritis (RA) is a chronic disease of the joints characterized by leukocyte infiltration, progressive destruction of articular structures, and degeneration of the cartilage. It is reported that an abnormal autoimmune response is important in RA development, but the exact causes of RA are still unknown. CD38 is expressed on various hematological tissues, such as B cells, NK cells, and monocytes [21], and the CD38 gene may regulate a variety of diseases. For example, the increased expression of the CD38 gene is related to poor prognosis in chronic granulocytic leukemia. Moreover, the impaired immune system caused by CD38 gene deficiency is also accompanied with poor prognosis. However, the function of the CD38 gene in DCs still remains unclear. The well-established experimental system used to study RA is the CIA model, in which heterogeneous CII proteins are used in the presence of an adjuvant to stimulate antisynovial autoimmunity. To test the effects of CD38 gene deficiency on RA, we employed this model as done previously [22].

DCs are specialized cells that link the innate and adaptive immune systems. The involvement of DCs in autoimmunity has been reported in patients as well as in animal models. When activated, DCs upregulate costimulatory molecules, produce cytokines, promote the priming of T cells, and activate various types of immune cells. Without activation, DCs show a low antigen presentation ability that might lead to unresponsiveness of T cells and play an important role in promoting immune tolerance [23, 24]. Silencing of DCs results in suppression of the immune response both *in vivo* and *in vitro*. The mature DCs express high levels of MHC II, T cell costimulatory molecules on the plasma membrane, and proinflammatory cytokines [25]. Tol-DCs have been developed as a cellular therapy, where T cell proliferation and IL-2 production were inhibited. NF-*κ*B is a transcript family which plays a vital role in regulating immune responses; the subunits of NF-*κ*B include RelA, RelB, c-Rel, p50/p105, and p100/p52 [26, 27]. RelB appears to be a key molecule that regulates the maturation, differentiation, and functions of DCs [13, 14, 26]. Shih et al. reported that siRNA-mediated silencing of RelB expression radically altered the DC maturation process and resulted in blunted antigen-specific T cell responses *in vitro* and *in vivo* [13]. Wu et al. reported that little T cell stimulating capacity is shown in splenic DCs of RelB mutant mice [14, 26]. We reported that the silencing of IL-12 [7] or RelB [4] in DCs leads to the expansion of Treg cells and tolerance induction [4, 7]. In this experiment, compared with WT CIA mice, the expression of RelB in CD38^−/−^ CIA mice decreased significantly; this reduction might influence BMDCs and lead to alleviate inflammation. In other studies, we demonstrated that *ex vivo* silencing of CD40 in DCs can ameliorate RA [28] and allergic diseases [29]. In this study, we demonstrated that the expression of the RelB gene is significantly decreased, accompanied with the inhibited maturation of DCs in CD38^−/−^ mice.

As discussed above, the involvement of DCs in tolerance and autoimmunity is bidirectional. DCs are professional APCs which have a potent ability to activate naive T cells and promote effective responses of T cell. DCs might also induce tolerance through the generation of Treg cells and through the inhibition of T cell responsiveness [30]. This dual function of DCs can be explained partly by the different maturation stages of DCs [31, 32]. T cell immune responses to self-antigens also play important roles in the development and ongoingness of RA. Supporting this possibility, data shows that CD38 gene deficiency is able to inhibit antigen-presenting function and CII-specific T cell responses. IL-10 is an anti-inflammatory cytokine and it can promote the tolerogenic function of DCs [33, 34]. Autoreactive T cells can cause an autoinflammatory immune response; however, the tolerogenic DCs show decreased ability in inducing an autoreactive T cell response. In this study, we suggested that the expression of the IL-4 and IL-10 genes is significantly increased, accompanied with the significantly decreased expression of IL-1*β* when the CD38 gene is deficient. However, compared with WT mice, the expression of TNF-*α* has no statistical difference. The reason might be that the mice used to set CIA models are usually DBA/1 (H-2^q^) mice, whereas the CD38^−/−^ mice we used in this study are C57BL/6 (H-2^b^) mice which are less sensitive to collagen II.

Phosphorylation of NF-*κ*B subunits leads to an increase in NF-*κ*B transcription activity and inducing production of cytokines. Inhibiting the expression or function of NF-*κ*B is crucial for maintaining DCs in an immature state [35–37], with the decreased expression of various cytokines, such as IL-1*β*, IL-6, and TNF-*α* [38, 39], which further amplify the inflammatory responses [40].

Tolerogenic DCs might also be induced either by promoting the expression of IL-4, IL-10, Fas, or TRAIL [41–43] or, conversely, by inhibiting the expression of immunostimulatory molecules [44, 45]. In this study, we identified that the phosphorylation of NF-*κ*B is greatly decreased in CIA mice when the CD38 gene is deficient. So, our results have shown that without stimuli, the phosphorylation of NF-*κ*B1 p105 shows no difference in the WT and CD38^−/−^ healthy mice, whereas, in the CIA mice model, the phosphorylation of NF-*κ*B1 p105 is CD38-dependent. In the current study, we generated a CIA model in both the WT and CD38^−/−^ mice. We have been able to demonstrate that the CD38 gene can be used as an effective target for suppressing autoimmune arthritis, although the mechanism behind this needs further identification. This finding could be used to develop new CD38-based therapies for autoimmune arthritis.

## 5. Conclusion

We demonstrate that the maturation and immunorepression of DCs were inhibited in CD38^−/−^ mice. We further delineate that the joint damage accompanied with suppression of proinflammatory cytokines attenuated, simultaneously, the phosphorylation of NF-*κ*B decreased in CD38^−/−^ CIA mice. This is the first demonstration that CD38 could be a potential target for the treatment of RA.

## Data Availability

The data used to support the findings of this study are available from the corresponding author upon request.
